# Synergy between Photodynamic Therapy and Dactinomycin Chemotherapy in 2D and 3D Ovarian Cancer Cell Cultures

**DOI:** 10.3390/ijms21093203

**Published:** 2020-04-30

**Authors:** Layla Mohammad Hadi, Elnaz Yaghini, Alexander J. MacRobert, Marilena Loizidou

**Affiliations:** Division of Surgery & Interventional Science, Faculty of Medical Sciences, University College London, London NW3 2QG, UK; elnaz.yaghini@ucl.ac.uk (E.Y.); a.macrobert@ucl.ac.uk (A.J.M.)

**Keywords:** actinomycin D, dactinomycin, ovarian cancer, photodynamic therapy, photochemical internalisation, combination treatment, 3D compressed collagen construct

## Abstract

In this study we explored the efficacy of combining low dose photodynamic therapy using a porphyrin photosensitiser and dactinomycin, a commonly used chemotherapeutic agent. The studies were carried out on compressed collagen 3D constructs of two human ovarian cancer cell lines (SKOV3 and HEY) versus their monolayer counterparts. An amphiphilc photosensitiser was employed, disulfonated tetraphenylporphine, which is not a substrate for ABC efflux transporters that can mediate drug resistance. The combination treatment was shown to be effective in both monolayer and 3D constructs of both cell lines, causing a significant and synergistic reduction in cell viability. Compared to dactinomycin alone or PDT alone, higher cell kill was found using 2D monolayer culture vs. 3D culture for the same doses. In 3D culture, the combination therapy resulted in 10 and 22 times higher cell kill in SKOV3 and HEY cells at the highest light dose compared to dactinomycin monotherapy, and 2.2 and 5.5 times higher cell kill than PDT alone. The combination of low dose PDT and dactinomycin appears to be a promising way to repurpose dactinomycin and widen its therapeutic applications.

## 1. Introduction

Photodynamic therapy is a minimally invasive modality for the treatment of solid tumours and certain non-cancerous lesions that relies on photoactivation of a photosensitising drug. Recently, the combination of chemotherapy or immunomodulation approach and photodynamic therapy (PDT) has been intensively investigated for the treatment of various types of cancers [1,2,3,4,5,6,7,8]. The rationale for the combination therapy approach is that the two modalities could be effective against different tumour sites thereby ensuring more effective treatment. For example, the PDT component can cause damage to the tumour vasculature, whereas the chemotherapy agent will act primarily against the tumour cells. Alternatively, PDT can aid delivery of the chemotherapeutic agents to the optimum sites in the tumour [9]. In addition to enhanced tumour destruction, it may also be possible to use a lower dose of the chemotherapeutic agent, thereby limiting any systemic toxicity.

**Figure 1:**
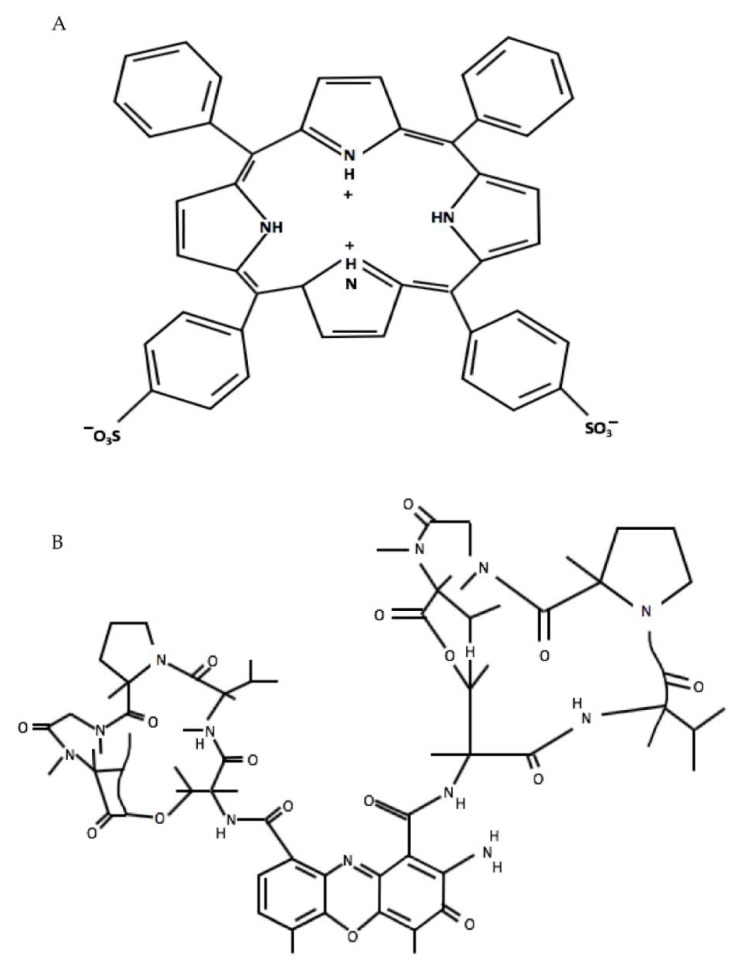
Structures of *meso*-tetraphenylporphine disulfonate (TPPS_2a_) (**A**) and dactinomycin (actinomycin D) (**B**).

A variant of light-activated combination treatment known as Photochemical internalisation (PCI) is an effective drug delivery system for the release of endolysosomally entrapped drugs into the cytosol. Entrapment or sequestration of biologically active agents that are taken up via endocytosis can reduce their efficacy since they cannot reach their intended intracellular targets and are subject to proteolytic degradation within the lysosomes [10,11]. In PCI, the photosensitisers used are amphiphilic and are located in endolysosomal membranes. Following light illumination, the photosensitisers are activated and generate reactive oxygen species (ROS), resulting in partial rupture of the endolysosomes enabling the sequestered agents to escape and reach their intracellular targets. The light and photosensitiser doses employed for PCI are relatively small corresponding to a low dose sub-lethal PDT treatment [12]. The amphiphilic photosensitiser, meso-tetraphenyl porphine disulfonate (TPPS_2a_), which has been employed in our study, bears two sulfonate groups on adjacent phenyl rings of the porphyrin macrocycle (Figure 1A) that can localise to the aqueous-lipid interface of lipid membranes with the unsubstituted more hydrophobic part of the macrocycle lying within the lipid bilayer [13]. These factors in addition to its photophysical properties enable the photosensitiser to be localised in the endolysosomal membrane and contribute to an efficient PDT and PCI effect [13,14,15,16]. Furthermore, an interesting feature of the disulfonated porphyrin-based photosensitisers that are typically used for PCI is that they are not susceptible to efflux by ABC transporters unlike certain other PDT photosensitisers. These transporters are one of the main sources of drug resistance to chemotherapy [6].

Various preclinical PCI studies have been shown to potentiate the biological activity of a large variety of molecules including gene-encoding plasmid, chemotherapeutic agents, liposomes, ribosome-inactivating proteins and adenovirus [5,6,10,11,17,18,19,20,21,22]. A further potential advantage is that neuronal toxicity is low [23]. The feasibility and safety of PCI has been recently demonstrated in a clinical trial for the treatment of head and neck cancers using bleomycin which is a hydrophilic glycopeptide agent with a molecular weight (MWt) of 1.4 kDa that is taken up into cells by endocytosis and is prone to entrapment and degradation in lysosomes [24].

In this study we investigated whether chemotherapy using another well-known agent, dactinomycin, could be potentiated for killing ovarian cancer cells in combination with low dose PDT. Ovarian cancer is one of the most frequently diagnosed cancers in women (almost 300,000 new cases worldwide in 2018) [25] but the treatment options are limited, which has stimulated the search for more effective therapies.

Dactinomycin or “actinomycin D” was the first antibiotic shown to have anti-cancer activity. It is a polypeptide antibiotic which is extracted from the genus *Streptomyces* [26]. Structurally it consists of a planar 2-aminophenoxazin-3-one chromophore and two cyclic pentapeptide lactones [27] (Figure 1B). Dactinomycin specifically intercalates into guanine-cytosine rich sequences, preventing RNA polymerase progression and eventually inhibiting the transcription process within cells [26,28]. Dactinomycin has been used as anti-cancer drug for the treatment of several cancers such as gestational trophoblastic cancer, testis cancer, Wilms’ tumour, rhabdomyosarcoma and Ewing’s sarcoma and ovarian cancer [26,29]. Both bleomycin and dactinomycin have been used for treating ovarian germ-cell malignancies [30]. For a chemotherapeutic, dactinomycin has a relatively high molecular weight of 1255 Da [31,32] and does not readily enter living cells. This molecular weight is comparable to bleomycin which is known to be very effective when used in combination with a low dose PDT regimen for PCI [33,34].

**Figure 2:**
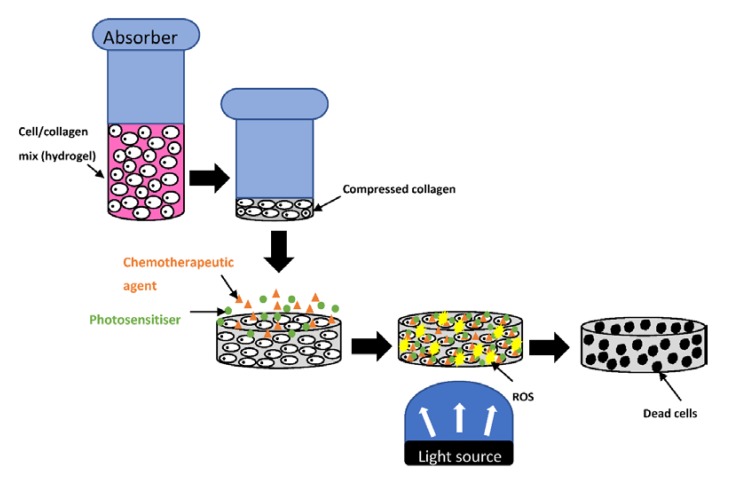
Combination treatment using photodynamic therapy and chemotherapy in a 3D compressed collagen cancer construct. The well containing Type 1 collagen hydrogel undergoes compression using an absorber to produce a higher density compressed collagen construct with a much lower water content. The photosensitiser (green) and chemotherapeutic agent (orange) are applied onto the construct and allowed to incubate before washing. The construct is then illuminated with light which results in generation of ROS (yellow).

The aim of the current study was to investigate the potential of dactinomycin for combination treatment for the first time in 2D cultures and 3D compressed collagen constructs of ovarian cancer cells, using TPPS_2a_ as the photosensitiser. 3D models offer better recapitulation of physiological properties than 2D and are becoming widely used in PDT [35,36]. Compressed type 1 collagen gels were used herein since they have a much higher density of collagen (c. 10% wt/vol) compared to standard hydrogels (<0.5% wt/vol), and therefore represent a more biomimetic construct and enable better cellular interaction with the extracellular matrix to be replicated. The lower rate of oxygen diffusion through higher density hydrogels is also relevant to PDT since it is reliant on an adequate supply of oxygen. Such properties place this 3D construct at an advantage compared to 2D models since this type of replication is not possible in monolayer cultures [37,38]. The cancer cells (HEY and SKOV3) selected for this study form papillary cystadenocarcinomas and clear cell adenocarcinomas respectively. Both of these cell lines develop in epithelial tissue which makes them suitable candidates for PDT and PCI treatment studies. Figure 2 shows schematically how the combination of photodynamic and chemotherapy is applied experimentally in the compressed collagen constructs.

## 2. Results

### 2.1. Combination Treatment and Phototoxicity Studies in 2D Cultures and 3D Constructs

**Figure 3:**
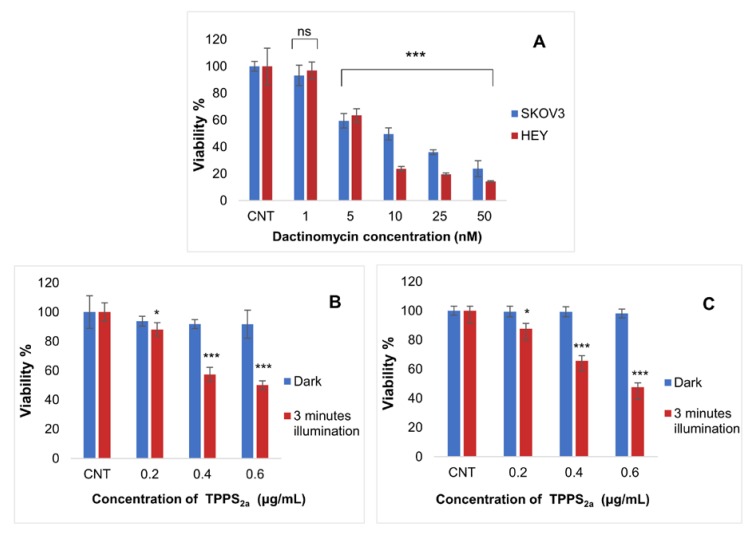
Toxicity of different dactinomycin concentrations using 24 h incubation on SKOV3 and HEY cell viability in monolayer culture (**A**) as well as viability of SKOV3 (**B**) and HEY (**C**) cells in monolayer cultures post PDT treatment using different concentrations of TPPS_2a_. For PDT the cultures were treated using a blue lamp at wavelength of 420 nM (light power of 7 mW/cm^2^) and total light dose of 1.26 J/cm^2^ (3 min). MTT was carried at termination stage. 1 nM is approximately 0.001255 µg/mL with the EC50 roughly being between concentration 5–10 nM which means that dactinomycin is highly toxic. Statistical analysis was carried out using 2-way ANOVA. * *p* < 0.05, *** *p* < 0.001, ns: *p* > 0.05. These *p* values show the significance difference between control and dactinomycin only treated cultures (histogram A) as well as between control and PDT treated cultures (histograms B and C).

In the first set of experiments we evaluated the efficacy of low dose PDT and dactinomycin chemotherapy on standard 2D monolayer cultures. The doses used for each treatment were sub-lethal. Figure 3A shows the dose response for dactinomycin only compared to control (CNT) sample in each cell line. The HEY cells appeared to be more susceptible to the drug. The dose dependence is in good agreement with a previous study on a different ovarian cancer cell line [39] and a wild-type polarized canine kidney cell line (MDCKII) [40].

For the combination treatment studies, we selected sub-lethal doses of the dactinomycin so that any potential synergy with PDT would become evident (Figure 3A). From preliminary PDT studies, it was determined that the correct photosensitiser doses to achieve sublethal phototoxicity (approximately 35% phototoxicity) using the lowest illumination period of our study (3 min) were 0.3 μg/mL and 0.4 μg/mL for SKOV3 and HEY cells respectively in both 2D (Figure 3B,C) and 3D cultures (data not shown). The dark toxicities caused by TPPS_2a_ at the chosen concentrations were found to be negligible (Figure 3B,C). We also used the same combination treatment protocol as in our previous study using saporin where the photosensitiser and chemotherapeutic agent were co-incubated for 24 h prior to light exposure [41]. The cells were also washed in drug-free medium before light exposure to reduce photosensitisation of the cell membrane and minimise direct photodynamic damage to the cells.

**Figure 4:**
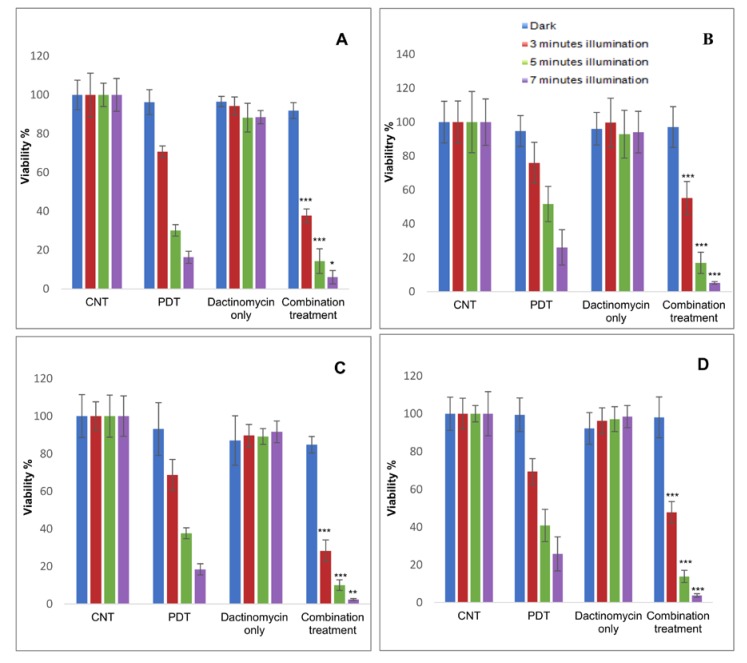
Percentage viability of SKOV3 (**A**,**B**) and HEY (**C**,**D**) cells in monolayer cultures after treatment with PDT, dactinomycin and combination treatment using Alamar Blue assay. The cells were treated with TPPS_2a_ (0.3 μg/mL for SKOV3 cells, 0.4 μg/mL for HEY cells), dactinomycin only (1 nM) and combination treatment. The cultures were treated using a blue lamp at wavelength of 420 nM and light power of 7 mW/cm^2^. The total light doses used were 1.26 J/cm^2^ (3 min), 2.1 J/cm^2^ (5 min) and 2.94 J/cm^2^ (7 min). The cultures were incubated for 48 h post illumination before terminating the experiment. Histograms A and C show results obtained from Alamar Blue assay and Histograms B and D show results obtained from MTT assay. Statistical analysis was carried out using 2-way ANOVA. * *p* < 0.05, ** *p* < 0.01, *** *p* < 0.001. These *p* values show the significance difference between PDT and combination treatments.

In both cell lines, with the 2D monolayer cultures a significant combination treatment effect and reduction in cell viability was achieved particularly compared to treatment with dactinomycin alone (Figure 4). Viability reductions from 94% to 38% (3 min), 88% to 14% (5 min) and 89% to 6% (7 min) vs. dactinomycin alone were observed in SKOV3 cells following measurements obtained with the Alamar Blue assay. The highest combined treatment efficacy (2.7- and 14.8-fold amplification in reduction of cell viability) was achieved after 7 min of light exposure compared to PDT and dactinomycin alone respectively. The corresponding alpha value for this irradiation period was 2.4.

A significant combined treatment effect in the HEY cells with the 2D monolayer cultures was also observed, where we found reduced viabilities from 90% to 28% (3 min), 89% to 10% (5 min) and 92% to 2% (7 min) compared to dactinomycin alone (Figure 4C, Alamar Blue assay). The amplification factors in treatment efficacy for combination therapy versus PDT alone and dactinomycin alone for the same data are 7.7- and 38.2-fold, respectively (*p* < 0.001). A good synergistic alpha value of 8.3 was also derived using this irradiation period. Table 1 shows a summary of the percentage viabilities, combined treatment efficacies and alpha values of the 2D monolayer cultures of both cell lines. There is generally good correlation between the Alamar Blue and MTT assay results.

**Figure 5:**
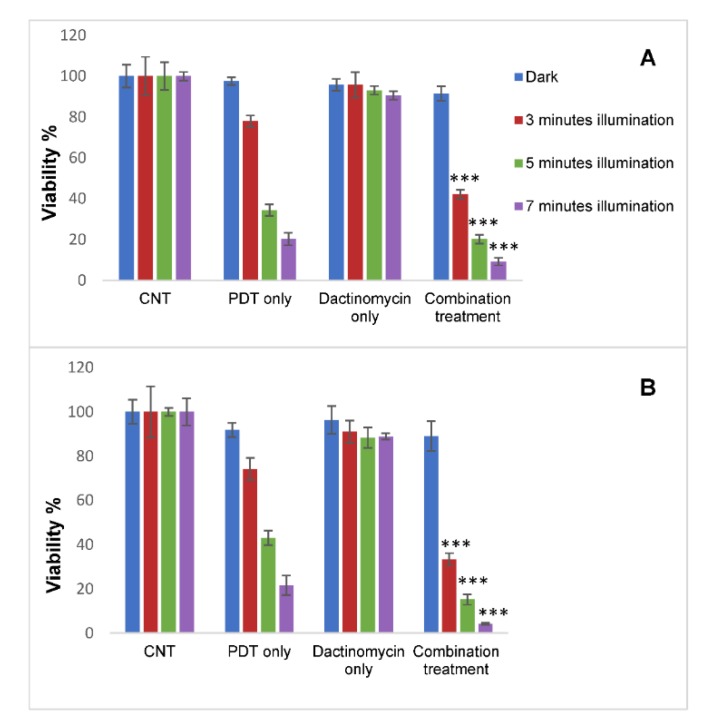
Percentage viabilities of SKOV3 (**A**) and HEY (**B**) cells in 3D compressed collagen constructs following PDT, dactinomycin only and combination treatments. The Constructs were treated with TPPS2a (0.3 μg/mL for SKOV3 cells) and (0.4 μg/mL for HEY cells), 1 nM dactinomycin and a combination of both drugs. The cultures were treated using a blue lamp at wavelength of 420 nm and light power of 7 mW/cm^2^. The total light doses used were 1.26 J/cm^2^ (3 min), 2.1 J/cm^2^ (5 min) and 2.94 J/cm^2^ (7 min). The cultures were incubated for 48 h post illumination before terminating the experiment. The results were obtained using Alamar Blue assay. Statistical analysis was carried out using 2-way ANOVA. *** *p* < 0.001. The *p* values show the significance difference between PDT and combination treatments.

In the 3D constructs a significant combined treatment effect was observed in both cell lines as shown in Figure 5 using the fluorimetric Alamar Blue assay. Since the constructs are strongly light-scattering the absorbance-based MTT assay is inappropriate for such studies. For example, in SKOV3 cells the highest combined treatment efficacies were attained after 7 mins of light irradiation, where the viabilities declined from 91% to 9% compared to dactinomycin alone and the efficacy values were 2.2 and 10.1 compared to PDT alone and dactinomycin alone respectively. The alpha value calculated for this condition was 2. Similarly, the HEY cells showed the highest reduction in viability (89% to 4%) and combination treatment efficacies compared to PDT alone and dactinomycin alone (5.5 and 22.3) after 7 min of light irradiation. For the same condition the alpha value calculated in this cell line was 4.9. The information described has been summarised in Table 2.

**Figure 6:**
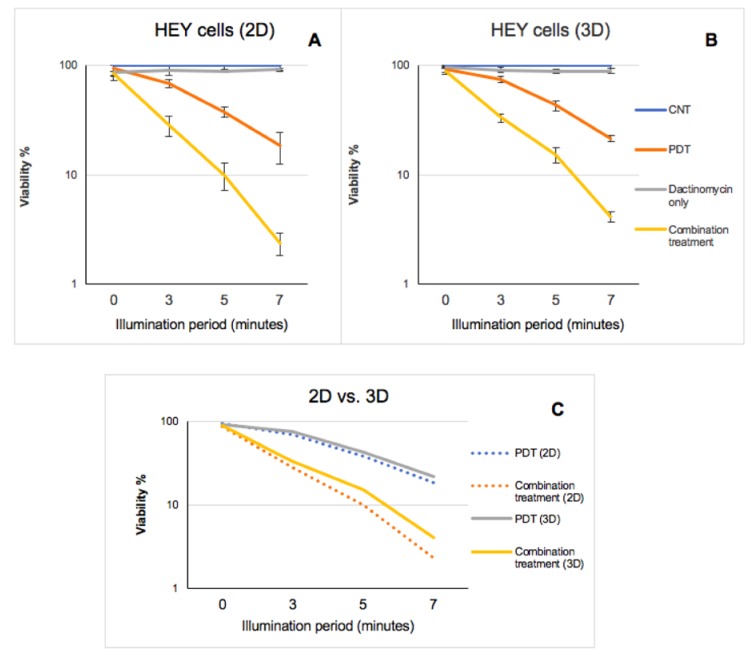
Semi-logarithmic graph comparing the effect of PDT, Dactinomycin only and combination treatment on viability of HEY cells in 2D (**A**) vs. 3D (**B**) cultures with increasing illumination periods. Graph (**C**) demonstrates a comparison of PDT and combination treatment effects on viability of HEY cells in each type of culture. The measurements were taken using Alamar Blue assay.

Although the reduction in percentage viability of both cell lines was slightly lower in 3D constructs compared to the 2D monolayer cultures e.g., 14% (2D) vs. 20% (3D) in SKOV3 cells and 10% (2D) vs. 15% (3D) in HEY cells, after 5 min illumination period, a significant cytotoxicity was still observed in both cultures (Table 1 and Table 2). Figure 6 shows the data for HEY cells in semilogarithmic form. As shown in Figure 6C, a much higher reduction in viability is observed post combination treatment compared to PDT in both culture models. Although both types of culture model demonstrate a similar pattern with regards to changes to the viability of HEY cells after each form of treatment, the effect of combination treatment is greater in the 2D cultures than the 3D cultures (Figure 6).

### 2.2. Live-Dead Viability Assay of 3D Constructs

**Figure 7:**
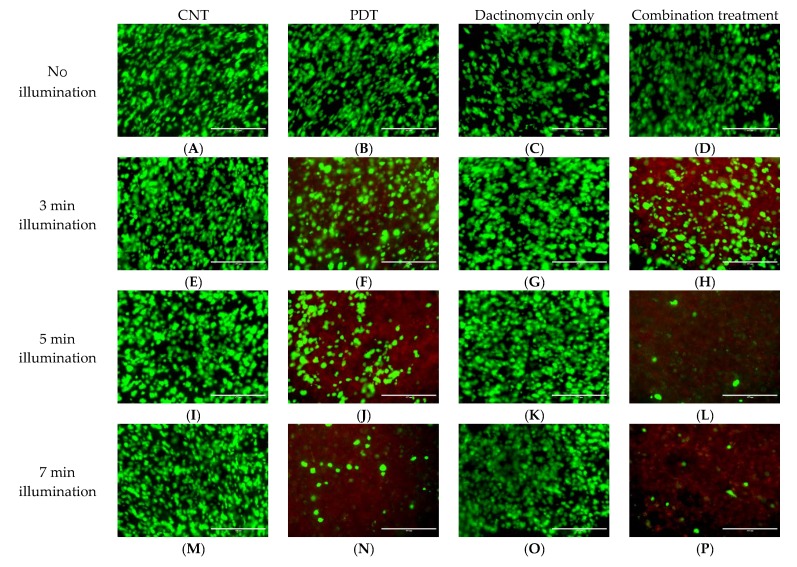
Live-Dead images of SKOV3 3D cultures post PDT (0.3 μg/mL) (**B**,**F**,**J**,**N**), dactinomycin only (1 nM) (**C**,**G**,**K**,**O**) and combination treatment (**D**,**H**,**L**,**P**) using different light conditions, and (**A**,**E**,**I**,**M**) without light. The cultures were treated using a blue lamp at wavelength of 420 nm and light power of 7 mW/cm^2^. The total light doses used were 1.26 J/cm^2^ (3 min), 2.1 J/cm^2^ (5 min) and 2.94 J/cm^2^ (7 min). Live-Dead assay was applied 48 h after illumination. To stain the live (green) and dead (red) cells, the 3D constructs were incubated with a solution containing Calcein-AM (live) and Ethidium homodimer-1 (dead). The scale bar presented in each image is 400 µm.

**Figure 8:**
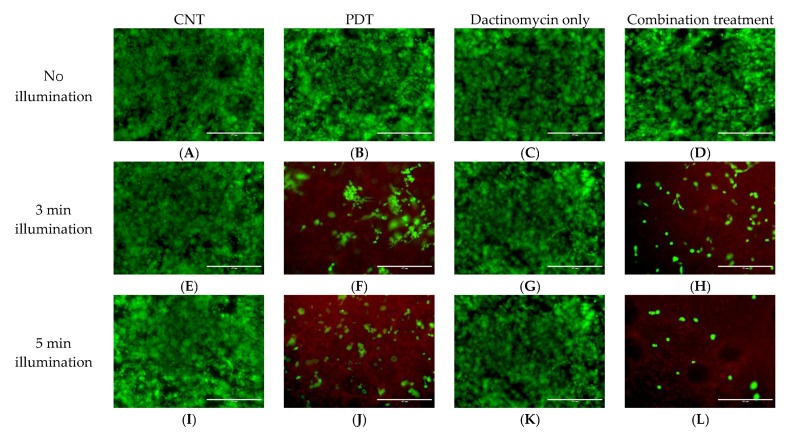

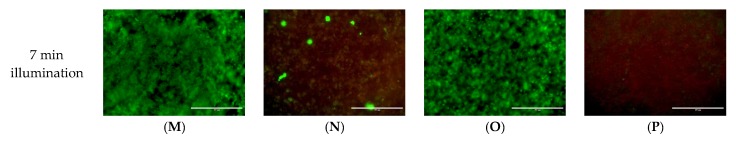
Live-Dead images of HEY 3D cultures post PDT (0.4 μg/mL) (**B**,**F**,**J**,**N**), dactinomycin only (1 nM) (**C**,**G**,**K**,**O**) and combination treatment (**D**,**H**,**L**,**P**) using different light conditions, and (**A**,**E**,**I**,**M**) without light. The cultures were treated using a blue lamp at wavelength of 420nm and light power of 7 mW/cm^2^. The total light doses used were 1.26 J/cm^2^ (3 min), 2.1 J/cm^2^ (5 min) and 2.94 J/cm^2^ (7 min). Live-Dead assay was applied 48 h after illumination. To stain the live (green) and dead (red) cells, the 3D constructs were incubated with a solution containing Calcein-AM (live) and Ethidium homodimer-1 (dead). The scale bar presented in each image is 400 µm.

The 3D constructs of both ovarian cell lines in this study were also subjected to Live-Dead staining to provide further evidence for the PDT and combined treatment effect. The live cells are presented with a green stain and the dead cells with red. Figure 7 and Figure 8 show live-dead images of SKOV3 and HEY 3D constructs following combination treatments using 1 nM dactinomycin and various illumination periods. The Live-Dead assay results support the findings from the cell viability analysis by showing the presence of a lower number of live cells after combination treatment compared to PDT or dactinomycin alone. Live-Dead images for samples treated with dactinomycin (2nM) only and combination treatment are shown in Figures S1 and S2. The control and PDT treated counterparts of the samples shown in Figures S1 and S2 are the same as those shown in Figure 7 and Figure 8, respectively, and are therefore not shown in SI. Figure S3 shows additional enlarged images of control and treated HEY cell 3D cultures after 5 mins light illumination.

## 3. Discussion

In this study we examined the combination treatment efficacy using the chemotherapeutic agent, dactinomycin, together with low dose PDT in two human ovarian cancer cell lines (SKOV3 and HEY) grown in standard monolayer culture vs. a 3D construct. Since the ECM consists mostly of structural proteins with type 1 collagen fibrils being the main component, a 3D compressed type 1 collagen construct was used as a platform for testing the treatments, as in our previous study using the cytotoxin saporin [41]. For oxygen-dependent treatments such as PDT, the much higher collagen density in the 3D construct compared to standard hydrogels is advantageous since oxygen diffusion is restricted in compressed collagen constructs owing to their higher density and collagen content (10% wt/vol compared to ~0.5% for uncompressed hydrogels) thereby recapitulating the restricted oxygen diffusion in tissue. According to Cheema et al. [42] the rate of oxygen diffusion is lower in compressed collagen gels by a factor of ten compared to uncompressed gels. This results in a lower equilibrium value of oxygen partial pressure and hypoxia within the compressed constructs since cellular oxygen consumption needs to be replenished by oxygen diffusion into the construct, as shown in previous work from our laboratory using pimonidazole staining for hypoxia [43].

The motivation for using a 3D construct model also stems from the known limitations of conventional 2D models which whilst presenting advantages such as cost effectiveness, simple preparation and maintenance and monitoring, cannot adequately integrate cellular-ECM interactions that exist in vivo since the cells grow on a flat surface, which also reduces cell to cell interaction [36,44,45]. Such restrictions affect the adhesion and organisation of cancer cells and may influence proliferation, signal transduction and response of cells to treatments [46,47]. Furthermore, the 2D cultures expose cells to a uniform environment with a steady oxygen and nutrient supply and facile drug diffusion which would normally be hindered in the presence of an ECM [48,49]. The incorporation of ECM in 3D models allows some of these limitations to be overcome enabling better replication of the in vivo environment [48,50,51,52]. However, 3D cultures also have disadvantages such as reproducibility between batches, complexity of matrix creation, extraction of cells for analysis, reduced sensitivity towards assays and screening instruments as well as potential difficulties with high-resolution imaging [37]. Even though our 3D platform is not able to fully mimic in vivo or clinical cancer models, the tunability of the components, and availability of different compartments allows for the creation of complex 3D cancer models that recapitulate many features observed in vivo as shown by Magdeldin et al. (2017) and Pape et al. (2019) [53,54].

In our study, TPPS_2a_ was chosen as a photosensitiser since it has been shown to be effective for the PCI technique [10,55,56], which is a combination treatment that relies on low dose PDT. A further advantage of this photosensitiser is that it is not a substrate for ABC transporters [18] which is an important consideration in combination treatment where the chemotherapeutic efficacy is often adversely affected by these efflux transporters.

We set out to demonstrate a synergistic interaction between dactinomycin and low dose PDT for enhancing cytotoxicity. We therefore selected sub-lethal doses of dactinomycin and TPPS_2a_. The dactinomycin toxicity data shown in Figure 3 are in agreement with results from previous studies on other cell lines, and we conclude that the cell lines used herein are not resistant to dactinomycin [40]. Cell viability was measured at 48 h post-light exposure which allows effects of apoptosis induced by dactinomycin to become apparent. According to studies by Lu et al. and Kleeff et al. dactinomycin is able to inhibit proliferation and induce apoptosis in pancreatic cancer and osteosarcoma cells [57,58].

By analogy with bleomycin which is being used in clinical trials for PCI and has a similar molecular weight at 1.4 kDa to dactinomycin, we hypothesized that dactinomycin might be effective with low dose PDT. This combination has not yet been explored previously in combination with PDT so far as we are aware. Studies by Shiraishi et al. (1986) and Zhitomirsky and Assaraf (2015) have provided indirect evidence for the lysosomal entrapment of dactinomycin which could occur via the ion-trapping mechanism for weak bases [59,60]. We used the same PCI protocol as in our previous study using 3D constructs [41].

The results of our study showed that the combination of sub-lethal PDT and a very low and non-toxic dose of dactinomycin was effective for enhancing cytotoxicity in two lines of ovarian cancer cells both in monolayer culture and 3D constructs. Using the longest illumination time for the 2D cultures, viability is reduced by 38.2 folds compared to dactinomycin alone in HEY cells (Figure 4C). Good correlation was seen between the fluorimetric Alamar Blue and MTT absorbance-based assay results for 2D cultures, as shown in Figure 4.

In 3D constructs of SKOV3 cells, the optimum combination treatment at 7 min resulted in a two-fold reduction in cell viability compared to PDT alone and a ten-fold reduction compared to dactinomycin alone as shown in Figure 5 and in the Live–Dead assay results (Figure 7). The combination treatment was found to be more effective in the HEY cell line than the SKOV3 cells for both 2D and 3D culture. These results are consistent with the dactinomycin cytotoxicity data shown in Figure 3A, where lower viabilities were measured for the HEY cells than SKOV3 for the same dactinomycin dose. We conclude that the combination treatment significantly amplifies dactinomycin cytotoxicity to comparable extents for each cell line. The synergistic interactions between PDT and Dactinomycin only treatment were calculated and presented in form of alpha (α) values (Table 1 and Table 2). An alpha value of above 1 corresponds to a synergistic effect whereas an alpha value below 1 corresponds to an antagonistic effect. From these calculations it was determined that synergistic interaction existed between the two therapies across all illumination periods in both 2D and 3D cultures of SKOV3 and HEY cells since all of the values calculated were above 1.5. In both 2D and 3D cultures of the two cell lines the highest alpha values were obtained after 7 min of light illumination. In 2D cultures the highest alpha values were found to be 2.4 and 8.3 in SKOV3 and HEY cells respectively whilst in the 3D cultures these values were found to be 2.0 and 4.9. The alpha values also showed that the HEY cells were more sensitive to the combination treatment than SKOV3 cells.

Dactinomycin is not readily taken up by living cells, and there have only been a few studies reported on the uptake mechanism. The uptake is known to be temperature sensitive and higher than expected for purely passive uptake via diffusion. Thus it may be mediated by uptake partly via fluid-phase endocytosis and thus suitable for PCI [61,62]. Alternatively an ion-trapping mechanism for retention in lysosomes, also known for doxorubicin [5] and other weak bases may apply [60]. Dactinomycin also contains primary and secondary amino groups and can therefore be protonated and charged at lysosomal pH which makes it more prone to entrapment since the ionised form is less likely to pass through the lysosomal membranes into the cytosol. Shiraishi et al. (1986) provided indirect evidence in human KB cells for the lysosomal entrapment of dactinomycin in a comparative study with another weak base doxorubicin (adriamycin) which is known to be subject to ion-trapping [59]. We have previously shown that PCI can be effective for enhancing doxorubicin cytotoxicity in doxorubicin-resistant breast cancer cells [5]. It is possible that the observed synergistic enhancement is in part due to a PCI mechanism but demonstration of endolysosomal localisation will be needed.

The more strongly fluorescent analogue of dactinomycin, 7-aminoactinomycin D, with essentially an identical molecular structure apart from the addition of an amino group, is used for staining dead cells. We attempted fluorescence imaging using 7-aminoactinomycin D to image the intracellular distribution, but the detection sensitivity was insufficient given that the compound is also cytotoxic like dactinomycin. There has been a previous report investigating the use of a delivery system for dactinomycin. Gronewald et al. found that co-administration with a cell penetrating peptide (CPP) enhanced cytotoxicity in MCF-7 breast carcinoma cells. The CPP was found to be taken up via endocytosis [63].

In our previous study using the same 3D construct model, the anti-cancer drug saporin was used to treat monolayer and 3D cultures of SKOV3 and HEY cells via PCI using the same illumination periods as the current study. The concentrations of TPPS_2a_ used were the same as this study, and the saporin concentration required to cause a significant combination effect was also very low in the nM range [41]. In that study, we attributed the enhancement observed using the combination treatment to the PCI mechanism, as others have concluded on the basis of light-induced redistribution of fluorescently-labelled saporin into the cytosol [55].

Interestingly, in both of our studies the HEY cells showed more sensitivity towards the combination treatment using dactinomycin or saporin than the SKOV3 cells, either in 2D or 3D. Another similarity between our previous study [41] and the current study is that for the 3D constructs the viability following treatment was slightly higher than the monolayer cultures (Table 1 and Table 2 and Figure 6). Such differences may be due to the collagen matrix in 3D constructs restricting cellular drug uptake due to reduced rates of diffusion through the matrix, which renders our 3D constructs closer to in vivo models than the monolayer cultures. The Live-Dead imaging studies are in good correlation with the Alamar Blue assay results. Fewer live cells (green) and more dead cells (red) can be observed in constructs exposed to combination treatment compared to PDT treated constructs which corresponds well with the Alamar Blue assay results. Furthermore, the population of live cells (green) decreases with increasing illumination periods.

A few studies have investigated PDT combination therapy in 3D cancer models as summarised previously [36]. Rizvi et al. [64] studied the combination of benzoporphyrin derivative PDT with carboplatin using an ovarian micronodule cancer model and found a synergistic enhancement of chemotherapy with PDT. Celli et al. [51] used the same model to analyse the response to benzoporphyrin derivative PDT in combination with a range of chemotherapeutics. The incubation times used prior to light exposure for the chemotherapeutics were longer (up to 72 h) than this study and a much shorter incubation time of 1 h was used for the benzoporphyrin derivative sensitiser, which is similar to that used in animal studies for this photosensitiser. Such differences make direct comparison with this study and our previous study [41] difficult, but they found the combination therapy was effective in nodule disaggregation, with paclitaxel showing the best efficacy. In another type of combination study by Lee et al. (2018), ultrasound was used with co-delivery by a microbubble system of a chemotherapeutic drug, doxorubicin, and a photosensitiser, chlorin e6, to pancreatic cancer cells in vitro and in vivo. An enhanced delivery efficiency of both therapeutics to a murine tumour model using ultrasound was observed in the in vivo studies [65]. Bazylinska et al. (2019) also investigated the co-delivery of cisplatin (chemotherapeutic drug) and photosensitiser, veteporfin, co-encapsulated in poly(lactide-co-glycolide) PLGA-based nanocarriers to SKOV3 ovarian cancer cells. The delivery of the drugs was either carried out using PDT or a combination of electroporation and PDT. The results showed that combining electroporation and photodynamic therapy to co-deliver the drugs led to a significant decrease in the viability of the ovarian cancer cells in a short time and enhanced the cell killing [66].

PDT alone and combination treatment using PDT have not been used in a clinical setting for the treatment of ovarian cancer to date. The findings of this study provide evidence of the effectiveness of these treatments in early simple in vitro models of ovarian cancer so these results can be applied to designing more advanced and complex in vitro 3D models for translation to preclinical in vivo studies. Based on other work from our laboratory, it should be possible to develop a complex compressed collagen ovarian cancer model consisting of cancer cells, fibroblasts, endothelial and mesothelial cells using our platform [53,54]. Nevertheless in vivo testing using preclinical models will still be required, particularly to establish the damage and healing response of normal tissue to the therapy. In future, our 3D platform or a modified version thereof could be used with patient tumour biopsy samples so that the response of the clinical tumour to PDT and different combination treatments can be pre-screened to enable personalised cancer treatment, instead of subjecting the patient to different combination treatments [67].

## 4. Materials and Methods

### 4.1. Cell Culture

Cell lines used in this study were Human ovarian carcinoma cell lines (SKOV3 and HEY) which were acquired from American Type Culture Collection (ATCC, London, UK). Medium DMEM/F12 (Sigma Aldrich, Dorset, UK) which was supplemented with 10% FBS (Thermo Fisher Scientific, Hemel Hempstead, UK) and 1% penicillin (5000 units/mL) and streptomycin (5000 µg/mL) (Thermo Fisher Scientific) was used to culture the cells.

### 4.2. Manufacture of 3D Cancer Constructs

The RAFT 3D culture systems protocol (Lonza, Slough, UK) was used for the manufacturing of the 3D in vitro cancer constructs, in the manner that we previously described [53,68]. First the hydrogels were prepared using 10% 10× MEM (used as colour/pH indicator), 80% Rat Tail Collagen Type I (First Link UK Ltd. Custom Bio-Reagents, Birmingham, UK) and neutralisation with neutralising solution consisting of 1.65 M NaOH and 840 mM HEPES buffer solution (Thermo Fisher Scientific). The cells were dispensed into the collagen mixture at 75,000 cells/construct density at overall (cells and collagen mix) volumes of 240 µL per well in 96 well plates. The hydrogels underwent incubation at 37 °C for 15 min to set prior to being exposed to plastic compression using absorbers (Lonza), at room temperature for a further 15 min. The absorbers were then removed, and fresh medium was added before returning the 3D constructs into the incubator.

### 4.3. In Vitro PDT/Combination Treatment Phototoxicity Studies in 2D Cultures and 3D Constructs

PDT and combination treatments were carried out in 2D monolayer cell culture for the purpose of comparison with the 3D construct. First the HEY and SKOV3 cells were seeded at densities 5000 (2D) and 75,000 (3D) cells/well in 96 well plates for 24 h. Concentrated stock solutions of the disulfonated tetrapenylporphine photosensitiser, TPPS_2a_ (Frontier Scientific Inc., Carnforth, UK), were prepared in dimethyl sulfoxide (DMSO) (3.3 mM). Preliminary studies were conducted on each cell line in order to determine the appropriate concentrations of the TPPS_2a_ and the chemotherapeutic agent, dactinomycin. Cells were incubated separately with either TPPS_2a_, in HEY cells at 0.4 µg/mL and for SKOV3 cells at 0.3 µg/mL, or dactinomycin (Sigma-Aldrich) at 1 nM or 2 nM for 20 h. Another set of cells were co-incubated with dactinomycin and TPPS_2a_ using the same concentrations for combination treatment. Afterwards the cells were washed with PBS before being incubated with drug and photosensitiser free medium for another 4 h. The cells then underwent illumination for up to 7 min using a blue LumiSource^®^ flatbed lamp with peak emission at 420 nm and 7 mW/cm^2^ output (PCI Biotech, Oslo, Norway). Measurement of the cell viability was obtained at 48 h after light illumination using the Alamar Blue assay. Assessment of control groups with no drugs added and with or without light exposure was also conducted.

### 4.4. Cell Viability Assay

Cell viability was determined using the Alamar Blue assay (Invitrogen, Thermo Fisher Scientific) and MTT assay (Sigma Aldrich). Briefly, for Alamar Blue assay solution, Alamar Blue dye was diluted in cell culture medium (10% of the total volume in cell culture medium) and added to the 2D or 3D cultures before being incubated at 37 °C for 4 h. After the incubation period, the supernatant from each well was transferred into black well plates. Fluorescence was measured using fluorescence plate reader (Fluoroskan Ascent, Thermo Labsystems, Philadelphia, PA, USA) at Ex/Em 530/620 nm. The Alamar Blue assay allowed the 3D models to be used for obtaining both quantitative and qualitative data since the assay is not toxic and therefore does not kill the cells. For MTT assay, MTT powder was dissolved in cell culture medium (1 mg/mL) and added to the cells. The cells were incubated with MTT solution for 2 h at 37 °C. The MTT solution was then removed from the cells and DMSO was added to the wells. The absorbance was measured at 525 nm using an absorbance plate reader (BioTek, Swindon, UK).

### 4.5. Live/Dead Staining for Fluorescence Imaging

The 3D constructs were stained for the purpose of viability imaging at pre-determined time points using the Live/Dead viability kit (Molecular Probes, Thermo Fisher Scientific). The 3D constructs underwent incubation with the Live/dead solution containing 0.05% of 4 mM Calcein-AM (λ_Ex/Em_ 495/515 nm) and 0.2% of 2 mM Ethidium homodimer-1 (λ_Ex/Em_ 495/635 nm) at room temperature for 30 min and were then imaged using an inverted fluorescence microscope (EVOS FL color, Life Technologies, Carlsbad, CA, USA).

### 4.6. Evaluation of Synergistic Effects

In order to determine whether a synergistic interaction exists between the two separate therapies applied, the value of alpha (α) was calculated using the following equation:

(1) $$\alpha \;=\frac{F_{PDT}\times F_{cytotoxin}}{F_{combination}}$$

The terms *F_PDT_* and *F_cytotoxin_* (shown in the numerator of Equation (1)) represent the fractional viability obtained after each separate therapy, PDT and the application of the cytotoxin. The denominator is the fractional viability observed following the PCI combination treatment. If α > 1 then a synergistic effect exists whereas an antagonistic effect is denoted by α < 1. Such analysis has been utilised previously by us and others to determine the presence of synergistic effects in PCI [69,70,71].

To assess the efficacy of the combination treatment against cytotoxin alone, we calculated the ratio of the viability without combination treatment (i.e., cytotoxin alone) divided by the viability measured after combination treatment. Likewise, to assess the combination treatment efficacy compared to PDT alone, the ratio of the viability was calculated by dividing PDT by the viability measured after combination treatment.

### 4.7. Statistical Analysis

We analysed the results using 2-way ANOVA with post-hoc analysis. Values of *p* < 0.05 were considered as statistically significant. The presentation of error bars from the mean indicates ± standard deviation (SD).

## 5. Conclusions

In this study we examined the efficacy of low dose photodynamic therapy in combination with dactinomycin as a chemotherapeutic drug in two human ovarian cancer cell lines, SKOV3 and HEY. Using different assays we found that the combination treatment was effective in both 2D and 3D cultures, although lower efficacies were observed in the 3D cultures. The better efficacy in 2D vs. 3D is a common feature of PDT studies with or without the incorporation of a chemotherapeutic agent which can be ascribed to multiple factors including diffusional restriction of the agents and oxygen. We also note that the disulfonated porphyrin employed herein has very similar intracellular localisation properties to a disulfonated chlorin that is currently in clinical trials [14]. For future studies, it will be interesting to see the effect of the combination treatment in complex 3D models of different cancers and in vivo. Tests should also be carried out on resistant cell lines as well as patient-derived primary cancer cells from different types of ovarian cancer for more clinical relevance. Moreover, dactinomycin could be used in combination with other photosensitisers to find out if similar results to this study can be achieved.

## Figures and Tables

**Table 1:** Summary of percentage viabilities ±% SD, combination treatment efficacies and alpha values in 2D monolayer cultures of SKOV3 and HEY cells. The concentration of Dactinomycin used was 1 nM. The cells were incubated for 48 h post light exposure before terminating the experiment. The measurements were obtained with Alamar Blue assay.

2D Culture								
Cell Line	TPPS_2a_ Concentration (µg/mL)	Light Exposure Period (Minute)	PDT only (% Mean Viability ±% SD)	Dactinomycin only (% Mean Viability ±% SD)	Combination Treatment (% Mean Viability ±% SD)	Combination Treatment Efficacy Ratio vs. PDT	Combination Treatment Efficacy Ratio vs. Dactinomycin only	Alpha Values
SKOV3	0.3	3	70.7 ± 2.9	94.3 ± 4.7	37.8 ± 3.5	1.9	2.5	1.8
HEY	0.4	3	68.7 ± 8.2	89.7 ± 5.9	28.4 ± 5.7	2.5	3.2	2.2
SKOV3	0.3	5	30.2 ± 3.0	88.2 ± 7.4	14.4 ± 6.4	2.1	6.3	1.9
HEY	0.4	5	37.7 ± 2.9	89.2 ± 4.2	10.0 ± 2.8	3.8	8.9	3.4
SKOV3	0.3	7	16.3 ± 3.1	88.5 ± 3.4	6.0 ± 3.4	2.7	14.8	2.4
HEY	0.4	7	18.4 ± 3.0	91.6 ± 5.8	2.4 ± 0.54	7.7	38.2	8.3

**Table 2:** Summary of percentage viabilities ±% SD, combination treatment efficacies and alpha values in 3D cultures of SKOV3 and HEY cells. The concentration of Dactinomycin used was 1 nM. The cells were incubated for 48 h post light exposure before terminating the experiment. The measurements were obtained with Alamar Blue assay.

3D Culture								
Cell Line	TPPS_2a_ Concentration (µg/mL)	Light Exposure Period (Minute)	PDT only (% Mean Viability ±%SD)	Dactinomycin only (% Mean Viability ± % SD)	Combination Treatment (% Mean Viability ± % SD)	Combination Treatment Efficacy Ratio vs. PDT	Combination Treatment Efficacy Ratio vs. Dactinomyin only	Alpha Values
SKOV3	0.3	3	78.0 ± 2.7	95.7 ± 6.3	42.2 ± 2.2	1.9	2.3	1.8
HEY	0.4	3	74.2 ± 5.0	91.1± 4.9	33.3± 2.8	2.2	2.8	2.0
SKOV3	0.3	5	34.4 ± 2.8	93.0 ± 2.1	20.1 ± 2.1	1.7	4.7	1.6
HEY	0.4	5	43.1 ± 3.2	88.3 ± 4.6	15.2 ± 2.4	2.9	5.9	2.5
SKOV3	0.3	7	20.3 ± 5.0	90.5 ± 2.9	9.2 ± 2.6	2.2	10.1	2.0
HEY	0.4	7	21.6 ± 4.4	88.8 ± 1.4	4.1 ± 0.4	5.5	22.3	4.9

## Supplementary Materials

The following are available online at ’https://www.mdpi.com/1422-0067/21/9/3203/s1, Figure S1: Live-Dead images of SKOV3 3D cultures post Dactinomycin only (2nM) and combination treatment using different light conditions, Figure S2: Live-Dead images of HEY 3D cultures post Dactinomycin only (2nM) and combination treatment using different light conditions, Figure S3: Live-Dead images of HEY 3D cultures post PDT (0.4 μg/mL) (B), dactinomycin only (1nM) (C) and combination treatment (D) and 5 minutes of light illumination.
