# Supplementary material for: Synergy between Photodynamic Therapy and Dactinomycin Chemotherapy in 2D and 3D Ovarian Cancer Cell Cultures
Source: Int J Mol Sci. 2020 Apr 30;21(9):3203. doi: 10.3390/ijms21093203 (PMC7247344; doi:10.3390/ijms21093203)
Supplement: Supplementary file 1 [file ijms-21-03203-s001.pdf]

Synergy between Photodynamic Therapy and Dactinomycin Chemotherapy in 2D and 3D Ovarian Cancer Cell Cultures

Supplementary Information

Layla Mohammad Hadi*, Elnaz Yaghini, Alexander J. MacRobert, Marilena Loizidou*

Division of Surgery & Interventional Science, Faculty of Medical Sciences, University College London, London NW3 2QG, UK

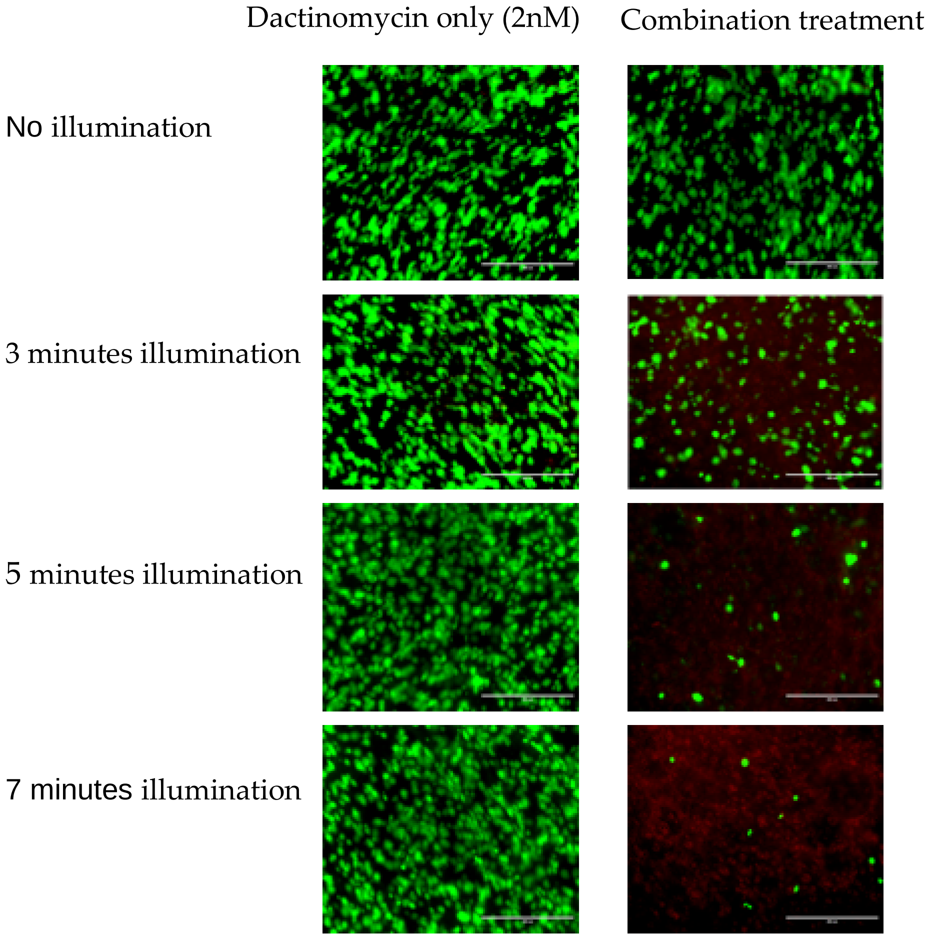

Figure S1: Live-Dead images of SKOV3 3D cultures post Dactinomycin only (2nM) and combination treatment using different light conditions. The control and PDT samples were same as those shown in Figure 5. The cultures were treated using a blue lamp at wavelength of 420nm and light power of 7mW/cm^2. The total light doses used were 1.26 J/cm^2 (3 minutes), 2.10 J/cm^2 (5 minutes) and 2.94 J/cm^2 (7 minutes). Live-Dead assay was applied 48 hours after illumination. To stain the live (green) and dead (red) cells, the 3D constructs were incubated with a solution containing Calcein-AM (live) and Ethidium homodimer-1 (dead). The scale bar presented in each image is 400 µm.

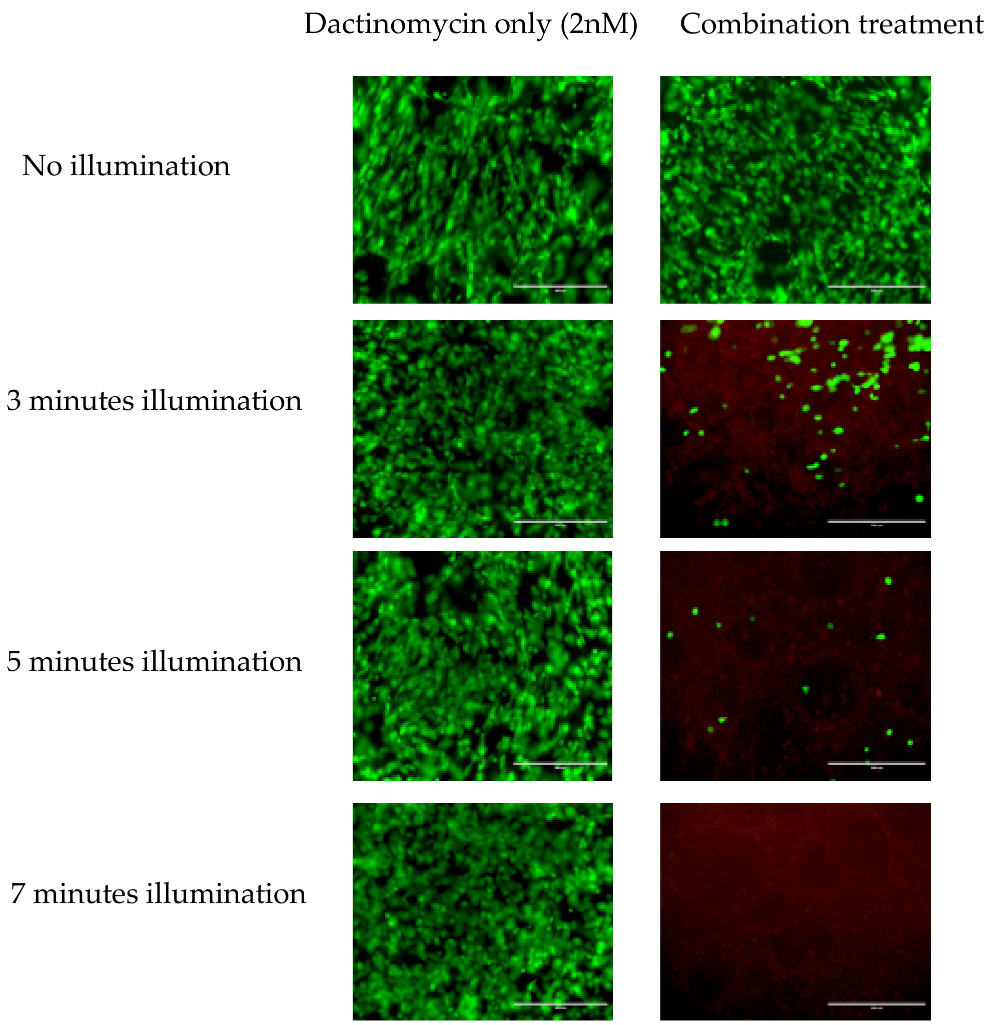

Figure S2: Live-Dead images of HEY 3D cultures post Dactinomycin only (2nM) and combination treatment using different light conditions. The control and PDT samples were same as those shown in Figure 5. The cultures were treated using a blue lamp at wavelength of 420nm and light power of 7mW/cm^2. The total light doses used were 1.26 J/cm^2 (3 minutes), 2.10 J/cm^2 (5 minutes) and 2.94 J/cm^2 (7 minutes). Live-Dead assay was applied 48 hours after illumination. To stain the live (green) and dead (red) cells, the 3D constructs were incubated with a solution containing Calcein-AM (live) and Ethidium homodimer-1 (dead). The scale bar presented in each image is 400 µm.

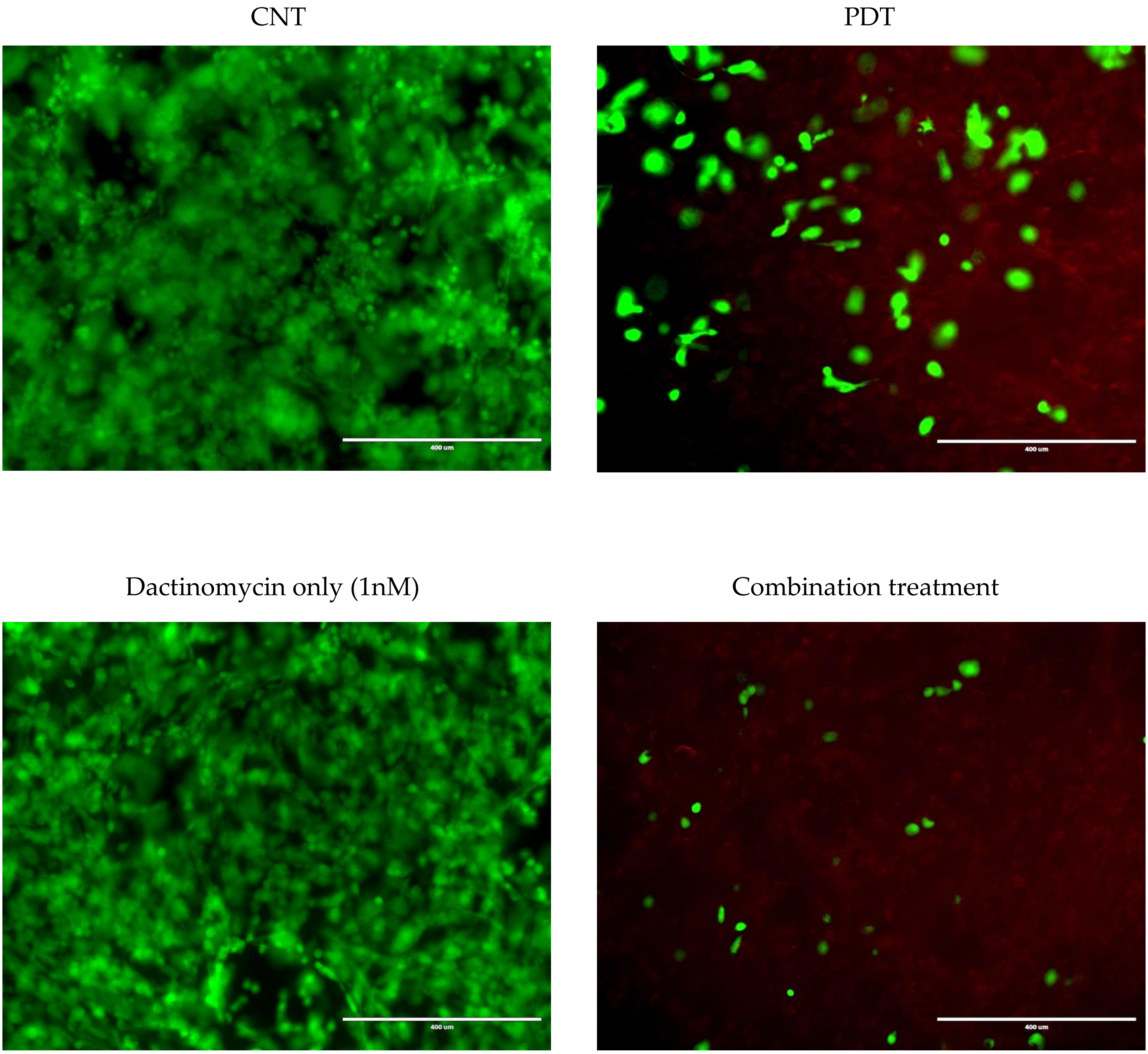

Figure S3: Live-Dead images of HEY 3D cultures post PDT (B), dactinomycin only (1nM) (C) and combination treatment (D) using 5 minutes of light illumination. The cultures were treated using a blue lamp at wavelength of 420nm and light power of 7mW/cm^2. The total light dose used was 2.10 J/cm^2 (5 minutes light illumination). Live-Dead assay was applied 48 hours after illumination. To stain the live (green) and dead (red) cells, the 3D constructs were incubated with a solution containing Calcein-AM (live) and Ethidium homodimer-1 (dead). Calcein represents the ubiquitous esterase activity in the live cells whilst Ethdium- homodimer 1 enters cells with damaged membranes and binds to nucleic acids within the cells. The scale bar presented in each image is 400 µm.
